# Why ‘publish or perish’? Why not ‘publish and prosper’? Perspectives from developing countries

**DOI:** 10.4103/0019-5545.46066

**Published:** 2005

**Authors:** Nimesh G. Desai

The exponential growth of science is too well known to require elaboration, although it is often not recognized that the growth is strongly linked to the phenomenon of scientific publi-cations. The printing and publication of scientific books, monographs and journals added impetus not only to the progress of science, but also to the interactive and sequential nature of scientific work. Recent electronic innovations have further transformed the field of scientific publication by enhancing storage, retrieval and enhancing access.

The growth of science coincided with the growth of competitiveness in many areas of human endeavour, leading to the need for comparisons and performance indicators. Whereas performance has been assessed by ‘production’ in the agricultural and industrial spheres, the indicator of performance in science—specifically academic science—became ‘publications’. The early trends in documenting scientific research for the genuine reason of disseminating information and thereby advancing the cause of science have been substituted by the imperative to publish for individual advancement. The pressure to publish as evidence of an individual's performance has steadily increased. In the characteristic late twentieth century western world, specifically in the American scenario, the dictum ‘publish or perish’ gained ground, reaching axiomatic relevance. The merits and demerits of this dictum have been discussed from time to time, but the realities in most developing countries such as India are so vastly different that a careful consideration in the national and regional context is essential.

The low rates of internationally published articles, the ‘inadequate’ or ‘poor’ quality of the articles published, and the lack of relevant research have often been commented upon.^1^–^3^ The unsatisfactory standards of biomedical journals published from India and the non-involvement of medical colleges in research have also been documented.^4^,^5^ It is generally known and accepted that the trends are similar for mental health research and this has also been occasionally documented.^6^ It has even been suggested, sometimes based on citation index and impact factor analyses, that the quality of scientific output from India has been declining as compared to other countries such as China, Thailand and the Philippines, and that developing countries should stop research because there is ‘much more to worry about than high quality research!’^7^,^8^ The despondency within, and the cynicism from without, presents a dangerous situation for academic scientific pursuits in the medical sciences, including psychiatry and mental health. There is a need to understand various aspects of the situation and find solutions to encourage research and ensure appropriate publication in international journals while raising the standards of Indian journals.

Fortunately, in the field of mental health, efforts at recognition of the need and crystallization of ideas for encouraging good and meaningful research, and appropriate scientific publication, have been occurring in the past few years.^9^,^10^ The need for documenting locally relevant experi-ence and research has also been emphasized.^11^

The difficulties and dilemmas of professionals who have chosen to work in the academic and/or public service sectors of India and other developing countries are worthy of attention. The lack of infrastructure and other facilities, the time and energy spent on routine clinical and training activities, alongside a less developed cultural tradition of ‘documentation’ all contribute to the publications being low in number and unsatisfactory in quality. Additionally, there is a lack of research training in medical schools, absence of incentives for being meaningfully productive in research and the possible ‘reaction formation’ of some professionals owing to the malpractices they see around them.

The fact that career promotion, in most settings, is not linked to research achievements in terms of publications, or for that matter almost any performance indicator, has been cited and discussed as one of the important contributing factors, besides the trends of part-time/honorary teachers in medical schools, and teachers with private practice. On the other hand, in a few settings with more academic leanings, the selection and career promotion process is linked to only the curriculum vitae—specifically publications. This has also been questioned since medical faculty positions, as a rule, involve multiple tasks—being a clinician, a teacher and a researcher, among occasional administrative assignments, all rolled into one.

It must be recognized that although the experience of western countries about the ill effects of the ‘publish or perish’ dictum may not be directly applicable in Third World countries, there can be a negative impact of the race for publication; the ‘rush’ to publish can easily lead to further dilution of the quality, and even compromise the basic ethical and scientific requirements. Committees overseeing promo-tion have occasionally been helpless regarding candidates whose publication record is astounding and almost unassail-able. Poor quality science is undesirable but dishonest science should be unacceptable!

The need for being watchful about violations or trans-gressions of ethical requirements is high, in view of the lack of standards or even awareness about these possibilities. The international experience with detection, investigation and prevention of fraud or dishonesty needs to be suitably adapted to our settings. It may be useful to remember that an excessive number of and/or ultrarapid publications by specific individuals have been commonly viewed with suspicion, especially if it is seen in the absence of an adequate back-ground in terms of funded research activities or clinical material.

The duties and responsibilities of editorial teams and boards include monitoring for ethical standards and detection of such irregularities but, more importantly, also pertain to encouraging research and publications, especially those relevant to the readership. It is worth highlighting that the long-standing tradition of mental health research in India, with pockets of high-quality and relevant research, needs to be actively promoted and streamlined. The strategies that can help are broadening the base of research interest and facilitating high-quality research publications of international standards. It cannot be overemphasized that these strategies will have to be coupled with the dilemmas of balancing scarce human resources in service delivery and research, develop-ment of public health systems and institutions versus individual career advancement, and increasing the quality of publications without diluting their quality further. The opportunities are therefore in terms of:

- Carrying out research relevant to local needs, with clinical and/or public health implications;
- Contributing to the international knowledge base on special topics such as the traditional concepts of mental health, spirituality, multiculturalism; and
- Evolving to take up a leadership role in the South Asian region and, possibly, for the rest of the developing world.

These opportunities, if availed of well, can contribute to the larger growth of science and development of nations.

The need of the hour is to stimulate research in developing countries, especially in mental health. The task of publishing in scientific journals is not only to be seen for career promotion in the western tradition, but also as a window to prospering academically and scientifically. The possibility of prospering, valid for teams and institutions, ultimately culminates in the prosperity of nations. Any implicit or explicit suggestion that developing countries can import the relevant science from the developed world with the economic and technological aspects, should be actively discouraged; and even the danger of passively falling prey to such an occurrence should be guarded against.

Why let situations of the nature of ‘publish or perish’ be created, with all its compulsiveness, ill effects and unethical nature? Why not let the healthy atmosphere of ‘publish and prosper’ be generated wherein the conduct of research and its publication is an end in itself and not a means to an end? The same healthy atmosphere needs to be generated for systematic documentation of clinic- or community-based experiences. The western dictum of ‘publish or perish’ needs to and can be effectively adapted to ‘publish and prosper’!
